# Mechanical Dentistry. Goodall's Patent Elastic or Spring Plate, for Partial Sets of Artificial Dentures

**Published:** 1869-02

**Authors:** E. B. Goodall

**Affiliations:** Portsmouth, N. H.


### ARTICLE III.

### *Mechanical Dentistry.*

### *Goodall's Patent Elastic or Spring Plate, for Partial Sets of Artificial Dentures.*

By E. B. GOODALL, Portsmouth, N. H.

The writer was much pleased with the article upon Mechanical Dentistry, by Dr. Reynolds, which appeared in the *Cosmos* for December last, and would heartily recommend it to the profession. "The Status of Mechanical Dentistry" has improved decidedly, even within the past year, but there is yet room for further and greater improvement, which will surely come through yet unknown and strange devices, while our motto shall ever be "Excelsior."

During the past two weeks the writer has visited, and personally invited the Demonstrators of Mechanical Dentistry of the several colleges of Baltimore, Boston, New York, and Philadelphia, to the examination of his invention, which is the subject of this article, and he here takes occasion to record his gratitude to these gentlemen for the open, cordial and intelligent character of his reception, and especially at the unbiased tone of their criticism, their frank acknowledgement of the novelty and the hearty approval of the merit of his spring plate for partial dentures.

At the suggestion of Dr. Gorgas of the Baltimore Dental College, to whom he demonstrated his method, he has prepared the followingi nstructions in detail, for the use of such of the profession as may avail themselves of that, which will give their patients more comfort and themselves more profit and satisfaction than any other branch in the line of their profession.

By this method every natural tooth can be retained, however irregular, provided only that it be properly plugged if decayed, or treated by appropriate remedies well known to the profession.

After obtaining an accurate impression of the mouth, either of wax or plaster, taking care not to draw down the wax around the natural teeth in removing the impression, proceed to make the plaster cast, *retaining all the natural teeth thereon*, and with a pencil draw a curved line around the entire arch of the plaster cast from a fourth to three-fifths of an inch from the necks of the natural teeth, usually terminating the plate at the proximal surfaces of the first and second molars where incisors or cuspidati re to be inserted, and extend the plate around to the posterior surface of the dens sapentiæ on each side. When bicuspids and molars are to be inserted, cut out from the plaster cast all the palatal portion to an eighth or a sixteenth inch of the pencil line before mentioned: thus forming and shaping the cast so that the plate when packed will be in two strips or bands joined firmly and neatly over the rugae, and when highly vulcanized there will be a spring to each strip or two elastic flexible bands, which are *practically automatic*, in the full meaning of the word. Of course the forms ( as to width and length,) is varied somewhat according to the number of teeth to be inserted, and according to the position and arrangement of the natural teeth. By scraping the lingual necks of the molars or the plastic cast one sixteenth of an inch, taking care to scrape only above the margin of the gum, or the second bicuspids, if the plate terminates there, the plate will be more secure, but this is not necessary unless the arch is shallow, and mucous membrane soft, spongy and springy. Ordinarily a perfect impression, and well packed rubber base, secures not only a *perfect adaptation*, but the plate is retained in place securely without suction or clasps, simply by its *elasticity*, as these spring plates do not rock or tip even when masticating upon artificial bicuspids or molars, on either side or both sides, and the same is true in regard to central and lateral incisors, either single, duplicate or all together. In his practice the writer has substituted with perfect success his spring plate on rubber base for all partial dentures and during the past year has inserted a large number of partial dentures for patients who formerly wore a clasp or suction plate, and has received the warmest and most sincere thanks and gratitude for the neat, light, and thin, spring plate substituted for a suction or clasp plate. He is aware that some dentists will be quite ready to say of this principle (as is the case with everything new and untried,) that it will press upon the natural teeth and produce soreness and discomfort, and is not applicable *to all cases*—the fact is (and *facts* are *very practical*) that partial dentures inserted by the method herein described, *do not* occasion *pain*, *soreness* or *discomfort* to the *natural* teeth, but this being the most plausible objection, it may be well to say that it is of no practical import, for suppose the plate to be heated over an alcohol lamp ten or fifteen seconds and the two bands to be spread one-sixteenth of an inch or more, the result would be, at first, an undue pressure upon the natural teeth, but in by one or two days at most, the pressure would be overcome the expansion of the arch so that the plate would be held in place by its elasticity or spring alone. If the strips were quite short and stiff, then there would be a continual wedging but the above method is essentially and practically different, and entirely automatic as before described.

Then again the palate being uncovered, speech and taste are unimpaired and all former objections are overcome. All the spaces and divisions between the natural teeth should be neatly waxed before packing, and when finished all the points between the natural teeth should be highly polished with the rest of the plate, making a complete and beautiful piece of work.

The writer has scores of affidavits and testimonials from highly educated and reliable parties, but will here mention only one case, that of a dentist of fine reputation and good practice, who has had seven partial dentures, inserted by as good and practical dentists as any of our large cities can boast, with only indifferent success, having recently adopted this new spring plate on rubber base, he says "I am pleased with the improved plan you have invented for partial dentures, and am confident that it will meet a want long and sorely felt by the profession. After many unsuccessful attempts (extending through several years,) by means of clasp or suction to have a partial set properly fitted to my mouth, I am now wearing one after your principle with great and increasing satisfaction." It is an acknowledged fact, that partial dentures have been heretofore regarded as rather discouraging and unsatisfastory in a majority of cases. Every practical dentist who desires the advancement of the profession will gladly welcome this great *desideratum* in mechanical dentistry, for its facility of construction, economy, perfect adaptability, and general utility.

The writer would be glad to illustrate by drawings from casts &c., but cannot do so now, but proposes to issue circulars to the dentists of the Unites States in due time.

